# Glioblastoma-Associated Mesenchymal Stem/Stromal Cells and Cancer-Associated Fibroblasts: Partners in Crime?

**DOI:** 10.3390/ijms25042285

**Published:** 2024-02-14

**Authors:** Thibault Lootens, Bart I. Roman, Christian V. Stevens, Olivier De Wever, Robrecht Raedt

**Affiliations:** 1 4Brain, Department of Head and Skin, Ghent University, 9000 Ghent, Belgium; thibault.lootens@ugent.be; 2 Laboratory of Experimental Cancer Research, Department of Human Structure and Repair, Ghent University, 9000 Ghent, Belgium; olivier.dewever@ugent.be; 3 Cancer Research Institute Ghent (CRIG), 9000 Ghent, Belgium; bart.roman@amalustherapeutics.com (B.I.R.); chris.stevens@ugent.be (C.V.S.); 4 SynBioC, Department of Green Chemistry and Technology, Faculty of Bioscience Engineering, Ghent University, 9000 Ghent, Belgium

**Keywords:** glioblastoma, tumor microenvironment, mesenchymal stem/stromal cells, cancer-associated fibroblasts

## Abstract

Tumor-associated mesenchymal stem/stromal cells (TA-MSCs) have been recognized as attractive therapeutic targets in several cancer types, due to their ability to enhance tumor growth and angiogenesis and their contribution to an immunosuppressive tumor microenvironment (TME). In glioblastoma (GB), mesenchymal stem cells (MSCs) seem to be recruited to the tumor site, where they differentiate into glioblastoma-associated mesenchymal stem/stromal cells (GA-MSCs) under the influence of tumor cells and the TME. GA-MSCs are reported to exert important protumoral functions, such as promoting tumor growth and invasion, increasing angiogenesis, stimulating glioblastoma stem cell (GSC) proliferation and stemness, mediating resistance to therapy and contributing to an immunosuppressive TME. Moreover, they could act as precursor cells for cancer-associated fibroblasts (CAFs), which have recently been identified in GB. In this review, we provide an overview of the different functions exerted by GA-MSCs and CAFs and the current knowledge on the relationship between these cell types. Increasing our understanding of the interactions and signaling pathways in relevant models might contribute to future regimens targeting GA-MSCs and GB-associated CAFs to inhibit tumor growth and render the TME less immunosuppressive.

## 1. Introduction

Glioblastoma (GB) is one of the most aggressive and most frequently occurring malignant primary brain tumors, representing more than 60% of all gliomas [1]. The cell of origin remains the subject of debate, but recent studies suggest that GB originates from neural stem cells (NSCs), NSC-derived astrocytes or oligodendrocyte precursor cells [2]. NSCs have self-renewal and proliferative capacities and are located in the subventricular zone of the brain. They can migrate to other regions, leading to the development of malignant gliomas by acquiring driver and additional mutations [3].

According to the 2021 World Health Organization (WHO) classification, GB is defined as an isocitrate dehydrogenase (IDH)-wildtype, adult-type diffuse grade IV glioma. Mutations in the TERT promotor region, epidermal growth factor receptor (EGFR) gene amplification and copy number variations in chromosome +7/−10 are frequently observed as well [4,5]. Based on molecular characteristics, three subtypes are defined in GB: the classical, proneural and mesenchymal subtypes [6]. Interestingly, a transition of the classical subtype into the mesenchymal subtype is observed in almost 50% of patients with IDH-wildtype gliomas going into a recurrent state. Higher levels of stromal, immune and myeloid cells are found in these mesenchymal substates, associated with resistance to therapy and a poor survival outcome [7]. Tumor heterogeneity is characterized by the presence of four main cellular states, namely astrocyte-like, mesenchymal-like, oligodendrocyte-progenitor-like and neural-progenitor-like, which are influenced by the TME and exhibit plasticity based on in vivo lineage-tracing studies [8]. Inter- and intratumoral heterogeneity, the complex TME and the blood-brain barrier (BBB) contribute to a poor median overall survival of 14–16 months after diagnosis [1].

The standard of care consists of the maximal surgical removal of the tumor, followed by a combination of radiotherapy and chemotherapy with temozolomide (TMZ) [9]. This approach targets cancer cells, but neglects their complex interplay within the TME. Recent studies suggest that the simultaneous targeting of malignant cells and their ecosystem could counteract drug resistance and immune escape [10]. GB TME cells can be classified as (1) immune cells, such as tumor associated macrophages/microglia, natural killer cells, myeloid-derived suppressor cells (MDSCs), neutrophils and T cells, and (2) non-immune cells such as glioblastoma-associated mesenchymal stem/stromal cells (GA-MSCs), cancer-associated fibroblasts (CAFs), pericytes, endothelial cells, neurons and astrocytes [11,12].

**Figure 1:**
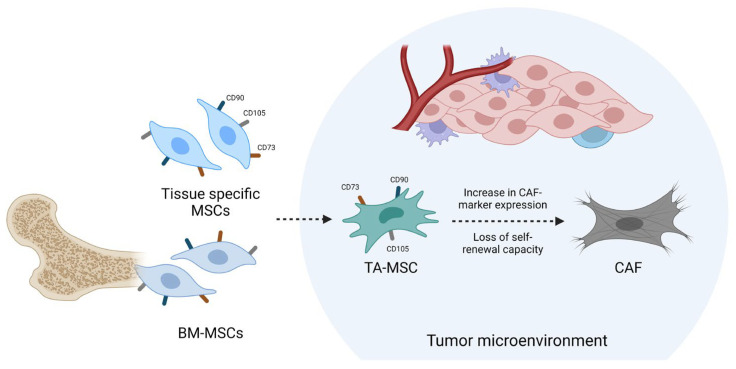
MSCs are recruited to the tumor microenvironment, where they differentiate into TA-MSCs, which subsequently transition into CAFs. This transformation involves a loss of self-renewal capacity and an increase in CAF-marker expression. BM-MSCs: bone-marrow derived mesenchymal stem cells; MSCs: mesenchymal stem cells; TA-MSC: tumor-associated mesenchymal stem/stromal cell; CAF: cancer-associated fibroblast. Created with BioRender.com.

Across multiple cancer types, tumor-associated mesenchymal stem/stromal cells (TA-MSCs) have emerged as an important cell type present in the TME [13,14]. In GB, these cells are able to enhance tumor growth and invasion, increase angiogenesis, stimulate GSC proliferation and stemness, mediate resistance to therapy and contribute to an immunosuppressive TME [15,16,17,18,19]. The current consensus states that bone-marrow derived mesenchymal stem cells (BM-MSCs) or tissue-specific MSCs are recruited to the tumor site, after which they acquire a tumor-promoting phenotype and differentiate into TA-MSCs [20,21,22]. MSCs are characterized by their self-renewal capacity and multipotency, as they are able to differentiate into osteoblasts, chondrocytes and adipocytes. They are found to be positive for cell surface markers CD90, CD105 and CD73 and negative for CD34, CD45, CD14 or HLA-DR, CD19 or CD79α and CD11b. In addition, MSCs must be plastic-adherent when cultured in vitro [23]. TA-MSCs share these features, but can be distinguished from MSCs by their altered secretome which results in a protumoral phenotype [24,25]. To differentiate TA-MSCs from MSCs, Coffman et al. developed a predictive algorithm based on RNA-sequencing data. Their classifier was able to successfully draw a distinction between these cell types in ovarian, endometrial and pancreatic cancer. Using the algorithm, they were able to differentiate TA-MSCs from CAFs as well [22]. According to current insights, TA-MSCs act as one of the precursor cells for CAFs, after losing their ability of self-renewal over time, along with an increased expression of CAF markers, as depicted in Figure 1 [13,14].

Due to the lack of fibroblasts in the healthy brain, CAFs were thought to be absent in the GB TME. Recent studies, however, accumulated evidence substantiating the existence of CAFs in GB [26,27]. Jain et al. isolated these cells from GB patient samples and performed an in-depth characterization using single-cell RNA sequencing (scRNA-seq) and spatial transcriptomics. In addition, a profiling of their functional effects on the tumor and the TME was performed. These cells had protumoral effects on GB stem cells (GSCs) and enhanced the polarization of macrophages towards a protumoral M2-like phenotype [26]. Tumor-associated macrophages (TAMs) can comprise up to 50% of the tumor mass and can be classified into two main phenotypes, namely the pro-inflammatory M1-like phenotype and the anti-inflammatory M2-like phenotype. Typical M1-like genes include *SPP1*, *CD74*, or *APOE*, whereas M2-like TAMs express *HLA-DR* and *CD163* [28]. However, recent studies revealed a more complex biology based on scRNA-seq of GB patient tumors, highlighting TAM heterogeneity and plasticity [29]. A more detailed exploration of the biology and functions of TAMs in the GB TME is provided elsewhere [28,29]. Another study by Galbo et al. provided an advanced bioinformatic analysis of scRNA-seq datasets of GB and revealed a transcriptomic profile of an outlier group of cells in accordance with the profile of CAFs from other solid tumors [27]. In addition, Zarodniuk et al. identified perivascular fibroblasts expressing CAF-associated markers in publicly available scRNA-seq datasets of GB, linked to poor survival outcomes and poor responses to immunotherapy [30]. Immunohistochemical staining confirmed the presence of perivascular fibroblast markers around blood vessels and in GB tumor tissue, while the expression of these markers was lower and limited to blood vessels in control tissue [31].

In this review, we will summarize the current knowledge on GA-MSCs and GB-associated CAFs and critically discuss the relationship between these cells. Expanding our knowledge about the complex interactions of GA-MSCs and GB-associated CAFs within the TME may contribute to the generation of novel therapeutics in the future.

## 2. TA-MSCs as CAF Precursors

In many cancer types, TA-MSCs are able to regulate immune responses through the production of metabolites and chemokines, enhance tumor progression, angiogenesis and metastasis and contribute to the development of resistance against therapy and an immunosuppressive TME. Furthermore, TA-MSCs alter the secretome of naïve BM-MSCs arriving at the tumor site, rendering them protumorigenic [25]. A comprehensive overview of the protumoral effects of TA-MSCs is provided elsewhere [13,14]. Interestingly, isolated TA-MSC populations can express CAF markers, suggesting an ongoing differentiation process towards CAFs [32,33,34]. Li et al. isolated TA-MSCs from lung cancer tissue, characterized by the expression of MSC markers and trilineage potential. An elevated expression of the CAF-related markers α-smooth muscle actin (α-SMA), HI-1α, MMP11, VEGF, CXCL12, TGF-β1, TGF-βRII, IL6 and TNFα was observed, suggesting differentiation towards a CAF phenotype [34]. Borriello et al. isolated a cell population expressing CAF markers S1004A, vimentin (VIM), fibroblast activation protein (FAP) and α-SMA, along with the expression of MSC markers CD73, CD90 and CD105, from neuroblastoma patient samples. As these cells were able to differentiate into osteoblasts, chondrocytes and adipocytes, they were named “CAF-MSCs”. These cells were able to increase the proliferation of tumor cells in vitro and enhance tumor growth in vivo [33]. After co-culturing BM-MSCs with human triple-negative breast cancer cells, Li et al. obtained TA-MSCs with the elevated expression of FAPα. They also showed that this enhanced FAPα expression mediated TAM recruitment and metastasis [32].

These results highlight TA-MSCs as an important protumoral cell type present in the TME and a potential cell of origin of CAFs.

## 3. Homing of MSCs and Differentiation into GA-MSCs

BM-MSCs hold great tropism towards tumor and wound-healing microenvironments [35]. Their migration capacity has also been demonstrated in in vitro and in vivo glioma models [36,37]. For example, Nakamizo et al. injected human bone marrow-derived MSCs into the internal carotid arteries of nude mice which had previously been intracranially implanted with GB cells. Based on fluorescent microscopy, BM-MSCs were found to selectively migrate towards these tumors [37]. In general, the homing of MSCs to injured sites can be ‘non-systemic’, when MSCs are already present in the target tissue, or ‘systemic’, when they have to reach these tissues after circulating through the bloodstream. The latter consists of a multistep process involving a wide variety of molecules. In both homing types, MSCs migrate towards the lesion guided by growth factors and chemokines [38]. TGF-β-treated MSCs showed a higher migratory capacity towards GB cells in vivo, based on fluorescent microscopy after injecting both cell types into the left striatum of nude mice. The mechanism behind this can, in part, be explained by the elevated expression of CXCR4 in MSCs after TGF-β treatment [39]. Furthermore, GB cells secrete stromal-derived factor-1 (SDF-1/CXCL12), which plays a crucial role in the recruitment of BM-MSCs into the tumor blood vessels [40]. Matrix metalloproteinase 1 (MMP1) is involved in the process of MSC migration, as both knockdown of MMP1 RNA and blockage of its cognate receptor PAR1 significantly reduced migration towards glioma-conditioned medium [41]. GB cells secrete hepatocyte growth factor (HGF), known as a chemotactic factor for MSCs. Neutralizing HGF secretion almost completely abrogated the migration of MSCs towards GB spheroids [42].

The mechanism behind BM-MSC differentiation into GA-MSCs remains unclear. One potential explanation involves the purinergic signaling molecule adenosine. The treatment of BM-MSCs with adenosine resulted in an altered secretome, fostering the invasion and proliferation of GB cells in vitro [43]. In melanoma, IL-17 may drive the differentiation of BM-MSCs into TA-MSCs in combination with IFN-γ via NF-κB signaling [44]. In addition, BM-MSCs may be differentiated into TA-MSCs via tumor-derived exosomes or tumor necrosis factor [13]. Upcoming research will be crucial to unraveling the mechanism behind this differentiation process in GB. GA-MSCs might originate from other sources as well, such as tissue-specific MSCs, which should be addressed in future lineage-tracing studies [22,45].

Notably, Hossain et al. reported GSCs as the origin of one of the isolated GA-MSC populations, based on an identical copy number and structural variations [16]. The differentiation of GSCs into GA-MSCs could therefore be considered a rare event, which requires further investigation in the future.

## 4. GA-MSCs Modulate the Tumor Microenvironment

### 4.1. Tumor Growth and Invasion

GA-MSCs enhance tumor growth and invasion through different mechanisms: (1) CXCL14, expressed by GA-MSCs, is positively correlated with upregulation of long non-coding RNA UCA1 and downregulation of miR-182 in GB cells, which drives glycolysis and tumor cell invasion through the UCA1/miR-182/PFKFB2 axis [46]. (2) CCL5 expression by GA-MSC is negatively associated with patient survival and CCL5/CCR5 signaling results in a higher invasion of GB cells and GSCs in vitro [47]. (3) GA-MSCs secrete C5a, which binds to its receptor expressed by GB cells and promotes GB invasion through the p38 MAPK-ZEB1 signaling pathway [15]. When C5a binds to its receptor on GA-MSCs, hyaluronic acid is produced in an autocrine manner, which promotes GB cell migration [48]. (4) CCL2 activates the expression of JAK1 and MLC2 in GA-MSCs, resulting in ECM remodeling and GB cell invasion [49]. (5) GA-MSCs showed the increased expression of Lysyl oxidase (LOX) and COL1A1, key factors in ECM remodeling, following co-culture with GB cells. This upregulation appears to be driven by the interaction between CD40L, derived from GB cells, and its receptor CD40 on GA-MSCs. This interaction triggers the translocation of NF-κB2 into the nucleus of GA-MSCs, resulting in elevated LOX expression [50]. Interestingly, the expression of COL1A1, but not LOX, by GA-MSCs was increased upon exposure to astrocyte-conditioned medium, suggesting a role of these glial cells in the process of GA-MSC-mediated ECM remodeling as well [50]. While these studies offer valuable mechanistic insights into the tumor-promoting effects of GA-MSCs, it is essential to assess the clinical relevance of GA-MSC targeting in future studies using suitable animal models, as discussed further on.

Extracellular purinergic signaling plays a pivotal role in GB and is linked to poor survival outcomes. The key molecule in this process is adenosine. In the TME, ATP and ADP are hydrolyzed by the enzyme CD39 to AMP, after which the latter is converted into adenosine via CD73. A recent single-cell spatial analysis revealed CD73 to be primarily expressed by tumor cells, while microglia were found to be the main source of CD39. Moreover, CD73^+^ tumor cells and CD39-expressing myeloid cells are spatially clustered, resulting in local adenosine hotspots [51]. These increased adenosine levels could result in an altered secretome of BM-MSCs, as IL-8 and TGF-β levels were significantly higher after treatment of BM-MSCs with adenosine. Moreover, conditioned medium from these adenosine-treated BM-MSCs significantly enhanced the proliferation and invasion of U343MG cells. Collectively, these findings suggest a role of adenosine in the crosstalk between GB cells and BM-MSCs, which should be elucidated in future research [43].

### 4.2. Angiogenesis

Angiogenesis is a crucial process in the development and growth of GB [52]. The co-injection of GA-MSCs with U87-MG cells into nude mice increased angiogenesis through the secretion of HGF and SDF-1/CXCL12 by these cells [53]. The intracranial xenotransplantation of GSCs into nude mice was associated with the higher expression of CD31, a marker of microvessel formation, larger tumor volumes and decreased survival if these GSC were derived from co-cultures with GA-MSCs versus GSC monocultures [17].

### 4.3. GSC Crosstalk

The co-injection of GA-MSCs and GSCs results in a higher chance of glioma formation and a larger tumor volume. GA-MSCs release IL-6, which binds to its coreceptor gp130 on GSCs, which activates the JAK-STAT3 pathway, resulting in increased stemness and the proliferation of GSCs. Interestingly, GA-MSCs increase the expression of mesenchymal markers in GSCs, which is linked to poor survival outcomes, as stated before [7,16]. GSC proliferation and self-renewal are also mediated by the uptake of GA-MSC-derived exosomes containing miRNAs. MiR-1587 mediates downregulation of the nuclear hormone receptor corepressor-1 (NCOR1) in GSCs, resulting in an increased proliferation [54].

### 4.4. Mitochondrial Transfer and Resistance to Therapy

Tunnelling nanotubes (TNTs) have been observed in connections not only between GB cells, but also between GB cells and astrocytes, as well as between GB cells and neurons. They promote tumor growth, initiation, angiogenesis and resistance to therapy, making them an intriguing therapeutic target [55,56]. Recently, Nakhle et al. showed the transfer of mitochondria from MSCs to GSCs via TNTs, resulting in enhanced energy metabolism and GSC proliferation. GSCs acquired resistance to TMZ through the transfer of mitochondria [18]. Next to TNTs, extracellular vesicles (EVs) are also involved in mitochondrial transfer from GA-MSCs to GB cells. [57]. Resistance to TMZ therapy is also established via the secretion of IL-6 by glioma-associated MSCs isolated from GB, astrocytoma and oligodendroglioma, resulting in the overexpression of FOXS1 in GB cells, driving the epithelial-mesenchymal transformation (EMT) process [58]. The elevated expression of FOXS1 might also result in an increased infiltration of TAMs and regulatory T cells, based on a pan-cancer analysis [59].

### 4.5. Effects on the Immune Compartment

**Figure 2:**
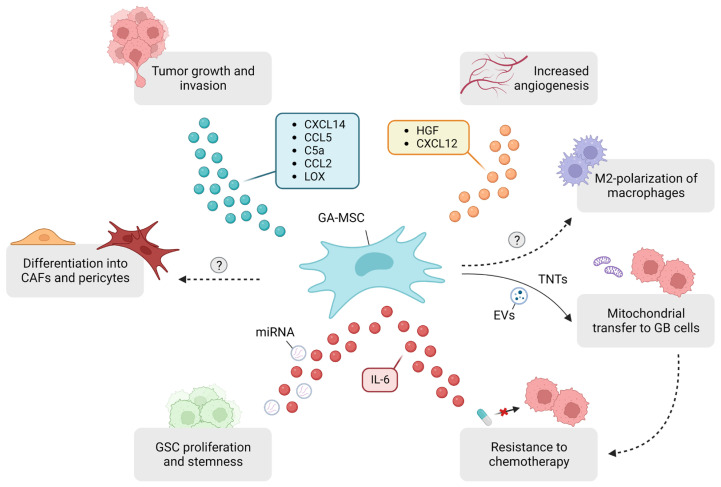
Schematic representation of GA-MSC interactions in the glioblastoma tumor microenvironment. Via the secretion of various factors, they are involved in important processes such as the promotion of tumor growth and invasion, via remodeling of the ECM matrix, increasing angiogenesis, stimulating GSC proliferation and stemness, mediating resistance to therapy, contributing to a ‘cold’ TME by macrophage polarization towards a protumoral phenotype and differentiating into pericytes and CAFs, which may exert protumoral effects as well. Question marks serve as indicators for the mechanisms that remain unknown. CAFs: cancer-associated fibroblasts; EVs: extracellular vesicles; GA-MSC: glioblastoma-associated mesenchymal stem cells; GSC: glioblastoma stem cells; TNTs: tunnelling nanotubes. Created with BioRender.com.

It is possible to discern the role of GA-MSCs in the establishment of an immunologically ‘cold’ TME, as Peng et al. observed a positive correlation between glioma-associated MSCs and a higher infiltration of regulatory T cells and M2-like macrophages. They co-grafted these cells with U87-MG cells intracranially into nude mice and performed transcriptome analyses on xenograft tumor tissues [19]. A cell-related gene signature was calculated based on differentially expressed genes between xenografts implanted with U87-MG cells alone and xenografts implanted with both U87-MG cells and glioma-associated MSCs [19]. When applied to patient data, this gene signature was correlated to overall survival, with a poor overall survival rate in patients with a higher score. In addition, patients characterized by higher expression levels of this cell-related gene signature had elevated stromal and immune scores, along with a lower tumor purity [19]. Xenografts co-implanted with U87-MG cells and glioma-associated MSCs showed larger tumor volumes compared to xenografts implanted with U87-MG cells alone, confirming the tumor-promoting effects of these glioma-associated MSCs in vivo [19]. As these cells were isolated from tissue samples of patients diagnosed with GB, anaplastic astrocytoma and anaplastic oligodendroglioma, and the authors did not specify the source of the TA-MSCs used in this study, future work will be crucial in order to elucidate the role of GA-MSCs on the GB immune compartment [19]. A summary of the effects established by GA-MSCs in the TME is depicted in Figure 2.

Although the percentage of TA-MSCs in high-grade gliomas is found to be inversely correlated with overall survival, the fractions of GA-MSCs in tissues from three GBs with a wild-type *IDH1* mutation status were only 0.7, 3.7 and 9.3% [60]. As the aforementioned studies incorporated higher ratios of GA-MSC in the in vivo models, future work should validate previous findings regarding the protumoral effects of GA-MSCs, taking into account their low abundancy.

## 5. GA-MSC Subpopulations

Two GA-MSC subpopulations have been defined based on the expression of CD90. CD90^-^ MSC-like cells produced significantly higher levels of the angiogenic factor VEGF compared to their CD90^+^ counterparts, suggesting a functional difference as well [61]. Differences in tumor-promoting effects and the secretion of growth factors and cytokines between CD90^high^ and CD90^low^ CAFs have also been reported for other tumors, such as prostate cancer [62]. Clavreul et al. identified two additional GA-MSC subtypes based on differential expression of CAF markers α-SMA, PDGFR-β and FSP1 and different tumor-promoting abilities [63]. The difference in subtype identification might be related to the ongoing differentiation process of GA-MSCs into CAFs, as discussed below.

## 6. Differentiation of GA-MSCs into CAFs

### 6.1. CAF-Marker Expression in GB

Multiple studies have identified cells expressing surface markers associated with CAFs in the GB TME [64,65,66,67,68]. However, as no single marker is uniquely expressed by these cells, it is difficult to distinguish them from related cell types such as pericytes, which show considerable overlap in cell surface markers [69,70,71]. Trylcova et al. found GFAP^-^ cells expressing α-SMA and TE-7 in the TME of human GB samples using immunohistochemistry [64]. Balaziova et al. demonstrated the expression of TE-7 and α-SMA, along with the expression of PDGFR-β (CAF- and pericyte marker), in FAP^+^ cells isolated from GB patient samples [65]. TE-7 can be used to identify fibroblasts and myofibroblasts, but lacks specificity, as pericytes may be stained as well [72,73]. In addition, Li et al. described immunopositivity for PDGFR-β in the majority of FAP^+^ cells in human GB samples. Remarkably, no co-labelling of FAP and α-SMA was observed in this study. Based on bulk RNA-seq data analyses, they observed a positive correlation in mRNA levels of FAP, PDGFR-β and two pericyte markers, CD13 and CD248, suggesting a subset of these FAP^+^ cells could be GB-associated pericytes [67]. Lastly, Zhao et al. isolated CAF-like cells from GB tissues involved in GB ferroptosis resistance through the expression of TSP-4. As only α-SMA and FAP expression was assessed by the authors, a more profound characterization process is required to confirm CAF identity [74].

### 6.2. Identification of GB-Associated CAFs

At present, three studies support the presence of CAFs in GB. Jain et al. isolated a CAF cell population from GB patient samples through serial trypsinization and confirmed their CAF-like nature via transcriptional analyses. These cells were able to enhance GSC proliferation through the expression of osteopontin (OPN) and HGF, and drive the polarization of macrophages towards a protumoral phenotype, mediated by the expression of fibronectin (FN) and its extra domain A (EDA) splice variant. Furthermore, angiogenic effects were observed on endothelial cells upon exposure to CAF-conditioned medium [26]. A spatial transcriptomic analysis revealed a close proximity of CAFs to GB cells of the mesenchymal-like subtype and protumoral macrophages [8,26]. GB CAFs were defined as non-tumor cells, based on copy number variation (CNV) analyses, expressing typical CAF-associated markers such as α-SMA, COL1A1, FAP, TNC, PDGFR-α, PDGFR-β, PDPN, VIM and S100A4. In contrast, these cells did not express markers associated with epithelial cells (CD326), endothelial cells (CD31), immune cells (CD45), astrocytes (GFAP) and pericytes (NG2, RGS5) [26].

Likewise, Galbo et al. identified an outlier group of non-malignant cells expressing CAF markers α-SMA, VIM, LOX, CAV1 and a series of collagens in a publicly available GB scRNA-seq dataset. In contrast, this cluster showed no expression of markers associated with endothelial cells (CD31, VWF), immune cells (CD45), astrocytes (GFAP), macrophages (CD14, CSF1R), B cells (CD79A/B) and pericytes (RGS5). The authors conducted a principal component analysis on GB RNA-seq datasets, initially categorized into four groups according to either the proneural or mesenchymal subtype and high or low CAF scores. Interestingly, proneural low-CAF and mesenchymal high-CAF groups were enriched on opposing sides along the first principal component axis, suggesting a role of CAFs in the proneural-to-mesenchymal transition in GB. High CAF probability scores correlated to poor survival outcomes and the expression of ECM remodeling gene sets linked to migration and invasion. The authors validated these findings in vitro by showing that migration capacities in primary cultures of GB patient-derived cell lines (PDCLs) were enhanced in 3/3 PDCLs and invasion capacity in 2/3 PDCLs upon exposure to conditioned medium of commercially available GB CAF. CAF identity was validated by the expression of α-SMA and VIM, the lack of GFAP expression, and a proteomics analysis showing a correlation between GB CAFs, fibroblasts and CAFs derived from oral tongue squamous cell carcinoma [27]. As additional CAF-associated markers should be assessed to confirm their CAF-like nature, the in vitro results should be approached with caution. Moreover, these results are opposed to the findings of Jain et al., as conditioned medium from the isolated CAF population failed to induce protumoral effects on nonstem GB cells [26]. Future work will be crucial to confirming these findings.

**Figure 3:**
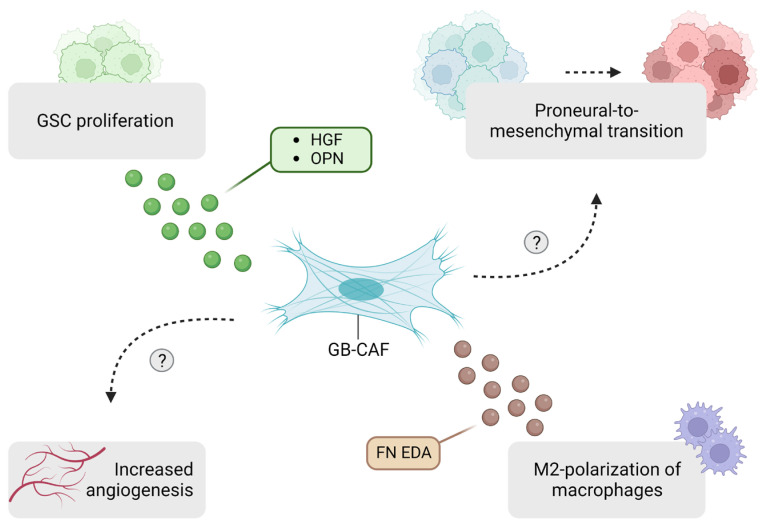
Schematic representation of the role of GB-associated CAFs in the glioblastoma tumor microenvironment. Question marks serve as indicators for the mechanisms that remain unknown. CAFs: cancer-associated fibroblasts; GSC: glioblastoma stem cells; HGF: hepatocyte growth factor; OPN: osteopontin; FN: fibronectin; EDA: extra domain A. Created with BioRender.com.

Zarodniuk et al. identified perivascular fibroblasts resembling CAFs in publicly available scRNA-seq datasets of GB, associated with a poor response to immunotherapy and a poor clinical outcome. Multiple CAF-associated markers such as PDGFR-α, PDGFR-β, COL1A1 and FAP were expressed by these cells, along with the CAF-specific transcription factor NR2F1. Importantly, these perivascular fibroblasts clustered separately from pericytes and smooth muscle cells. Interestingly, a CNV analysis identified a fraction of these cells as aneuploid, suggesting the presence of malignant cells with a phenotype very similar to CAFs, emphasizing the importance of a thorough characterization process [30]. In line with the findings of Jain et al., these perivascular fibroblasts are reported to recruit and polarize macrophages towards a protumoral phenotype, based on the analysis of intercellular communication networks from GB scRNA-seq datasets [26,30]. A schematic representation of the functions of GB-associated CAFs is depicted in Figure 3.

Taken together, these findings support the presence of CAFs in the TME of GB, where they might exert important functions, but some crucial questions remain unanswered: Are GA-MSCs one of the origins of CAFs, and if they are, what percentage differentiates into CAFs? Which mechanism could be responsible for this transition? Is it possible to develop a suitable animal model to study these knowledge gaps in vivo? How abundant are CAFs in the GB TME?

### 6.3. GA-MSCs as CAF Precursors

MSCs and TA-MSCs have been described as a potential source of CAFs in several cancer types [34,45,75,76]. The exposure of BM-MSCs to conditioned medium derived from glioma cells activates a transcriptional process resulting in cells expressing CAF-associated genes [75,77]. The factors involved in the differentiation of GA-MSC into CAFs remain elusive. Recent evidence suggests the incorporation of GSC-derived EVs as an important event, resulting in an altered secretion of exosomal miRNAs, and an activation into CAF-like cells [78]. The low abundancy of CAFs in public GB scRNA-seq datasets makes it hard to define their origin based on lineage trajectory analyses. Jain et al. performed these analyses on the expression data of GB CAFs and other stromal cells found in their serially trypsinized cultures. They excluded the possibility of CAFs arising from other stromal cells isolated by this technique, further supporting the hypothesis of GA-MSCs as a potential source of CAFs in GB [26]. Moreover, most of the perivascular fibroblasts described by Zarodniuk et al. clustered together with MSCs and expressed mesenchymal progenitor markers, indicating MSCs as a potential source of CAFs in GB [30]. Future lineage-tracing studies should be performed to confirm these results.

Besides the incorporation of EVs, TA-MSCs could lose their ability of self-renewal over time, resulting in the generation of a CAF cell population [13]. This may represent another possibility for the generation of CAFs from GA-MSCs, suggesting association with tumor progression. The expression of typical CAF markers α-SMA, PDGFR-β and S100A4, along with the expression of MSC markers CD73, CD105 and CD90, is reported for some GA-MSCs. In contrast, these GA-MSCs did not express markers associated with other cell types such as endothelial cells (CD31), astrocytes (GFAP), macrophages (CD14) and hematopoietic stem cells (CD34, CD45), and the expression of pericyte marker NG2 was observed in only a very small fraction of this population [46,79]. The latter is important, as typical CAF markers are also expressed by pericytes [70]. Conversely, several authors reported the expression of MSC markers, but not CAF markers, by GA-MSCs [15,16,49,54]. Besides, some of the CAF-marker-expressing GA-MSCs can differentiate into osteocytes, but not adipocytes [79].

**Figure 4:**
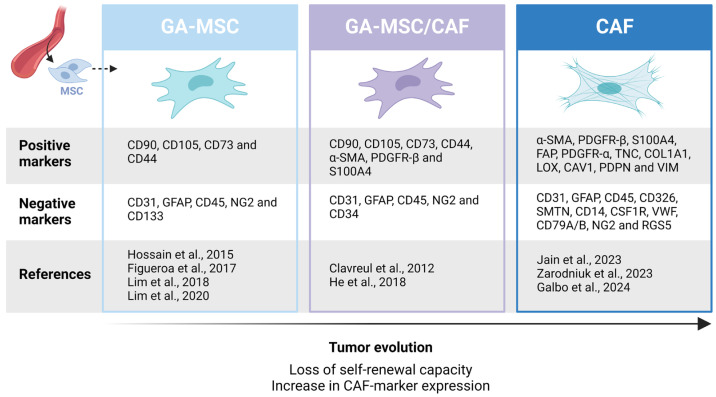
Differentiation process of GA-MSCs into CAFs during GB tumor progression [15,16,26,27,30,46,49,54,79]. Created with BioRender.com.

These findings could indicate a continuum where part of the GA-MSCs have a differentiation status closer to CAFs, while others are more closely related to BM-MSCs or tissue-resident MSCs. Such a concept is in agreement with the hypothesized time-depended differentiation of GA-MSCs into CAFs (Figure 4) [13]. Moreover, Madar et al. described CAFs as a cell ‘state’, rather than a cell type, further supporting the hypothesis of GA-MSCs differentiation ‘states’ enroute to a CAF state [80]. An important aspect of fortifying this hypothesis would be the evaluation of the loss of the trilineage potential in the isolated CAF population described by Jain et al. [26], something which should be addressed in future studies.

### 6.4. A Shared Origin with Pericytes

GA-MSCs may give rise to pericytes as well. First, Yi et al. reported an upregulated expression of pericyte markers and target genes involved in angiogenesis in GA-MSCs upon exposure to GB-conditioned medium [81]. Also, BM-MSCs are able to differentiate into pericyte-like cells when cultured in GB-conditioned medium [82]. Second, two studies reported the expression of pericyte markers NG2 and CD146 in a large proportion of GA-MSCs, indicating differentiation towards pericyte-like cells [53,63]. Third, immature MSC-like pericytes expressing CD90, CD248 and PDGFR-β have been identified in the GB TME. These cells were characterized by their ability to inhibit T cell proliferation and the expression of TGF-β, HGF and soluble human leukocyte antigen-G (sHLA-G), contributing to an immunosuppressive environment [83]. Collectively, these results indicate the possibility of a shared origin of pericytes and CAFs from GA-MSCs.

## 7. CAF Subtypes in GB

Due to the wide variety of functions, origins and markers, different CAF subpopulations have been described across many cancer types. Based on single-cell characterization, three main subtypes were identified: ‘Myofibroblastic CAFs’, ‘immune regulatory/inflammatory CAFs’ and ‘Antigen-presenting CAFs’ [84]. Another concept proposed by Simon et al. states the presence of four main states of CAFs, classified as ‘immune’, ‘desmoplastic’, ‘contractile’ and ‘aggressive’. A decrease in CAF heterogeneity is suggested over time, with contractile and aggressive states evolving at later stages [85]. A recent multiomic analysis reported three overarching clusters conserved across multiple cancer types and species, defined as steady state-like (SSL), mechanoresponsive (MR) and immunomodulatory (IM) CAFs. The interconversion of one supercluster into another has been reported, as immune checkpoint inhibitors caused a proportional shift with a lower proportion of SSL CAFs and a higher abundance of IM CAFs after treatment of basal cell carcinoma patients [86].

The heterogeneous expression of CAF markers is reported in GB, as Balaziova et al. observed the expression of α-SMA and PDGFR-β, in 59% and 88% of the isolated FAP^+^ GB mesenchymal stromal cells, respectively [65]. If these cells were indeed CAFs, these findings might correlate to breast cancer studies describing CAF subtypes that exhibit differential α-SMA, PDGFR-β and FAP expression. Breast CAFs exhibiting co-expression of α-SMA, PDGFR-β and FAP played key roles in immunosuppression, correlating to an immune regulatory subtype [84,87]. The hypothesis of the presence of CAF subtypes in GB has recently been addressed by Jain et al., who reported two CAF subtypes evolving from a more immature CAF subpopulation. In addition, they found gene expression patterns within the isolated cell population corresponding to the SSL, MR and IM substates described across cancer types [26,86]. Early and late-stage CAF subtypes seen in the isolated cell population were also observed in cell clusters with a high CAF probability score from publicly available GB scRNA-seq datasets. However, mainly the late stage subtype was present in this dataset [26].

As the isolated CAF population described by Jain et al. only emerged after 5 weeks of serial trypsinization, cell culture passaging could have induced changes in the expression of certain marker genes, possibly giving rise to culture-induced CAF subpopulations, or resulting in the loss of certain CAF subtypes present in the bulk tumor [69,88]. Therefore, CAF subtypes should ideally be defined before cell culture passaging. Due to their low abundancy, however, this will remain a challenging research question in the foreseeable future.

## 8. Open Questions and Future Perspectives

In recent years, GA-MSCs have been recognized as an important cell type in the GB TME. Multiple studies have demonstrated the effects of GA-MSCs on tumor growth and invasion, angiogenesis, GSC crosstalk and resistance to therapy [15,16,18,19,46,47,48,49,50,53,54,57]. Several of the mechanisms behind these processes remain unanswered and should be addressed in future research. An important aspect that warrants further investigation is their potential effect on the GB immune compartment. To date, Peng et al. showed a positive correlation between glioma-associated MSCs and a higher infiltration of protumoral macrophages [19]. As clinical advancements of immunotherapies in GB have been limited thus far [89], future studies should elucidate the effects of GA-MSCs on the immune compartment and evaluate whether targeting these cells could render immunotherapies more effective.

Future lineage-tracing studies will be crucial to unraveling the cell of origin and differentiation process producing GA-MSC. Despite the extensive range of protumoral functions and their link to poor survival outcomes, the scarcity of GA-MSCs might question the potential for future therapeutic strategies targeting these cells in GB [60]. This urges the need to evaluate future regimens in suitable pre-clinical models that account for this low abundancy.

CAFs are known as an important cell type in multiple cancers, and their recent discovery in GB highlights them as an emerging therapeutic target. As GA-MSCs and CAFs show considerable overlap in cell surface markers, active signaling networks and functions, GA-MSCs might be seen as CAF precursors, as proposed in this review. The alternative origins of CAFs should be explored in future research as well, given their high degree of heterogeneity and plasticity in other cancer types [90]. Culture conditions might impact this cell plasticity, along with marker expression [69,88], indicating that the presence and characterization of current GB-associated CAF subtypes should be approached with caution [26]. Emerging multiplex staining procedures could entail an interesting approach to validating these findings, as over 200 biomarkers may be studied simultaneously, providing spatial information as well [91,92].

Similar to GA-MSCs, GB-associated CAFs could be considered a rare cell type in the TME [27]. Jain et al. proved the ability of GB CAFs to promote tumor growth in vivo, by inoculating a ratio of 1:7 CAFs and GB neurosphere cells into the brains of athymic mice [26]. As this number is not representative of the number of CAFs observed in tissue-derived scRNA-seq data [27], this might bring into question the magnitude of effects observed in these co-implanted xenograft models. Studying these effects in genetically engineered mouse models for GB could bypass these limitations [26], as MSCs might be recruited to these naturally formed tumors, giving rise to GA-MSCs and CAFs in representative numbers. One example could entail the EGFR-driven genetic mouse model with the loss of CDKN2A and PTEN, reflecting the classical GB subtype [93]. As Yeo et al. performed scRNA-seq of both CD45^−^ tumor/non-immune cells and CD45^+^ immune cells derived from this model, future studies could reanalyze this dataset and look for outlier clusters resembling CAFs, similar to the approach of Galbo et al. [27,93]. As GB CAFs seem to be involved in proneural-to-mesenchymal transition [27], it might be interesting to validate their presence in PDGF-β-overexpressing and NF1-silenced murine tumors, resembling proneural and mesenchymal GB, respectively [94].

Finally, as no unique marker is known to be expressed by GA-MSCs and CAFs, a thorough characterization should be performed after cell isolation. Based on current literature, GA-MSCs could be defined as CD90^+^, CD105^+^, CD73^+^, CD44^+^, CD31^−^, GFAP^−^, CD45^−^, NG2^-^ and CD133^-^ cells with the capacity to differentiate into osteoblasts, chondrocytes and adipocytes [15,16,49,54]. GB CAFs, on the other hand, could be defined as α-SMA^+^, COL1A1^+^, FAP^+^, TNC^+^, PDGFR-α^+^, PDGFR-β^+^, PDPN^+^, VIM^+^, S100A4^+^, CAV1^+^, LOX^+^, CD326^−^, CD31^−^, CD45^−^, GFAP^−^, SMTN^−^, CD14^−^, CSF1R^−^, VWF^−^, CD79A/B^−^, NG2^−^ and RGS5^−^ cells [26,27,30]. The absence of their trilineage potential remains to be proven. Furthermore, for both cell types, demonstrating the lack of CNV and tumorigenic potential is crucial. Isolated cells that harbor characteristics from both GA-MSCs and CAFs could be considered intermediate states, as proposed in Figure 4.

## 9. Conclusions

GA-MSCs and GB-associated CAFs are highlighted as important cell types within the GB TME, with a potential time-dependent differentiation process of GA-MSCs into CAFs. Future research will be crucial to addressing the multitude of unresolved questions addressed in this review, which might contribute to future treatment strategies, and eventually enhance patient survival.

## Abbreviations

α-SMA	α-smooth muscle actin
BBB	Blood-brain barrier
BM-MSC	Bone marrow-derived mesenchymal stem cell
CAF	Cancer-associated fibroblast
CCR2	CC-chemokine receptor 2
ECM	Extracellular matrix
EDA	Extra domain A
EGFR	Epidermal growth factor receptor
EMT	Epithelial–mesenchymal transition
EVs	Extracellular vesicles
FAP	Fibroblast activation protein
FN	Fibronectin
GA-MSC	Glioblastoma-associated mesenchymal stem/stromal cell
GB	Glioblastoma
GSC	Glioblastoma stem cell
HA	Hyaluronic acid
HGF	Hepatocyte growth factor
IDH	Isocitrate dehydrogenase
IM	Immunomodulatory
LOX	Lysyl oxidase
MDSC	Myeloid-derived suppressor cell
MMP1	Matrix metalloproteinase 1
MR	Mechanoresponsive
MSC	Mesenchymal stem cell
NSC	Neural stem cell
OPN	Osteopontin
PDCL	Patient-derived cell line
RNA-seq	RNA sequencing
scRNA-seq	Single-cell RNA sequencing
SDF-1	Stromal-derived factor-1
SSL	Steady state-like
TAM	Tumor-associated macrophage
TA-MSC	Tumor-associated mesenchymal stem/stromal cell
TME	Tumor microenvironment
TMZ	Temozolomide
TNBC	Triple-negative breast cancer
TNT	Tunnelling nanotubes
VIM	Vimentin
